# Antitumoral effect of maintained neutrophilia induced by rhG-CSF in a murine model of pancreatic cancer

**DOI:** 10.1038/s41598-019-39805-y

**Published:** 2019-02-27

**Authors:** A. Brú, R. Bosch, M. V. Céspedes, S. Carmona-Güedes, E. Pascual, I. Brú, J. C. Souto

**Affiliations:** 1 Faculty of Mathematics, Universidad Complutense de Madrid, Madrid, Spain; 2 Laboratory of Oncology/Hematology and Transplantation, Institute of Biomedical Research, IIB Sant Pau, Barcelona, Spain; 3 Department of Hematology, Hospital de la Sant Creu i Sant Pau, Institute of Biomedical Research, IIB-Sant Pau, Barcelona, Spain; 4 Institut d’Investigacions Biomédiques Sant Pau, Hospital de Santa Creu I Sant Pau, Barcelona, Spain; 5 CIBER de Bioingeniería, Biomateriales y Nanomedicina (CIBER-BBN), Barcelona, Spain; 6 Centro de Salud La Estación, Talavera de la Reina, Spain; 7 Josep Carreras Leukemia Research Institute, Barcelona, Spain

## Abstract

Although the protumoral functions of polymorphonuclear neutrophils are well known, some now-forgotten studies report antitumoral roles for these cells. The present work examines the antitumoral effect of maintained neutrophilia induced via the injection of recombinant human granulocyte colony stimulating factor (rhG-CSF, 100 μg/kg/day) in a Panc-1 subcutaneous xenograft murine model of pancreatic cancer. This treatment was compared with gemcitabine administration (120 mg/kg every two days) and a saline control (n = 6–7 mice per group). Compared to the controls, both the rhG-CSF- and gemcitabine-treated mice showed significantly suppressed tumor growth by day 4 (p < 0.001 and p = 0.013 respectively). From a mean starting volume of 106.9 ± 3.1 mm^3^ for all treatment groups, the final mean tumor volumes reached were 282.0 ± 30.7 mm^3^ for the rhG-CSF-treated mice, 202.6 ± 18.1 mm^3^ for the gemcitabine-treated mice and 519.4 ± 62.9 mm^3^ for the control mice (p < 0.004 and p < 0.01, respectively, vs. control). The rhG-CSF-treated tumors showed higher percentage necrosis than those treated with gemcitabine (37.4 ± 4.6 vs. 7.5 ± 3.0; p < 0.001). This is the first report of a clear anti-tumoral effect of rhG-CSF when used in monotherapy against pancreatic cancer. Since rhG-CSF administration is known to be associated with very few adverse events, it may offer an attractive alternative in the clinical treatment of pancreatic cancer.

## Introduction

Pancreatic adenocarcinoma is an aggressive form of cancer^1^ that responds poorly to treatment. Most patients are diagnosed at an advanced stage, when over 50% already have metastases^2,3^. This cancer occupies fourth position in terms of overall mortality and first position in terms of mortality at 5 years (95%). Although numerous strategies have been designed to improve the effectiveness of current treatments, the results have not been greatly encouraging, with median overall survival ranging from 5 to 11 months^3^. Finding a successful treatment for this condition is one of the greatest challenges in oncology.

In recent years, immunological therapies based on the adaptive immune response have generated much interest as a means of fighting cancer. Chronic inflammation is commonly seen as a negative prognostic factor^4^, but there is growing evidence that acute inflammatory responses, involving mostly polymorphonuclear neutrophils (PMNs) and macrophages, help prevent the establishment and development of tumors^5^. Certainly, there is a qualitative difference between chronic and acute inflammation^6^. The former might be regarded as pro-tumoral given the accompanying degradation of the extracellular matrix around the tumor, which could allow for greater tumor growth and might also prevent tumor-seeking drugs from reaching their target. In maintained acute inflammation, however, the encapsulation of the tumor by neutrophils appears to have an antitumoral effect^4,6,7^. Indeed, a number of studies have shown that the critical factor affecting tumor growth is not the nutrient supply but the availability of space into which the tumor can grow^8^; when a tumor is encapsulated by neutrophils, the necessary space is already occupied by these immune system cells.

Numerous studies^4^ have shown that the administration of recombinant human granulocyte colony stimulating factor (rhG-CSF), which increases the number of circulating PMNs, has an antitumoral effect, probably via the above-mentioned mechanism. In humans, G-CSF is produced by many types of cell, including T cells, macrophages, endothelial cells and fibroblasts, upon receipt of the necessary stimulus. It then acts as a paracrine molecule that recruits neutrophils, monocytes and lymphocytes from the bloodstream to sites where they are needed. The aim of the present work was to examine the antitumoral effect of maintained neutrophilia induced by the administration of rhG-CSF in a xenograft murine model of pancreatic adenocarcinoma.

## Results

### rhG-CSF suppresses tumor growth in a xenograft model of pancreatic adenocarcinoma

A xenograft model of pancreatic adenocarcinoma using Panc-1 cells was generated in athymic mice to test and compare the antitumoral effect of rhG-CSF and gemcitabine.

**Figure 1:**
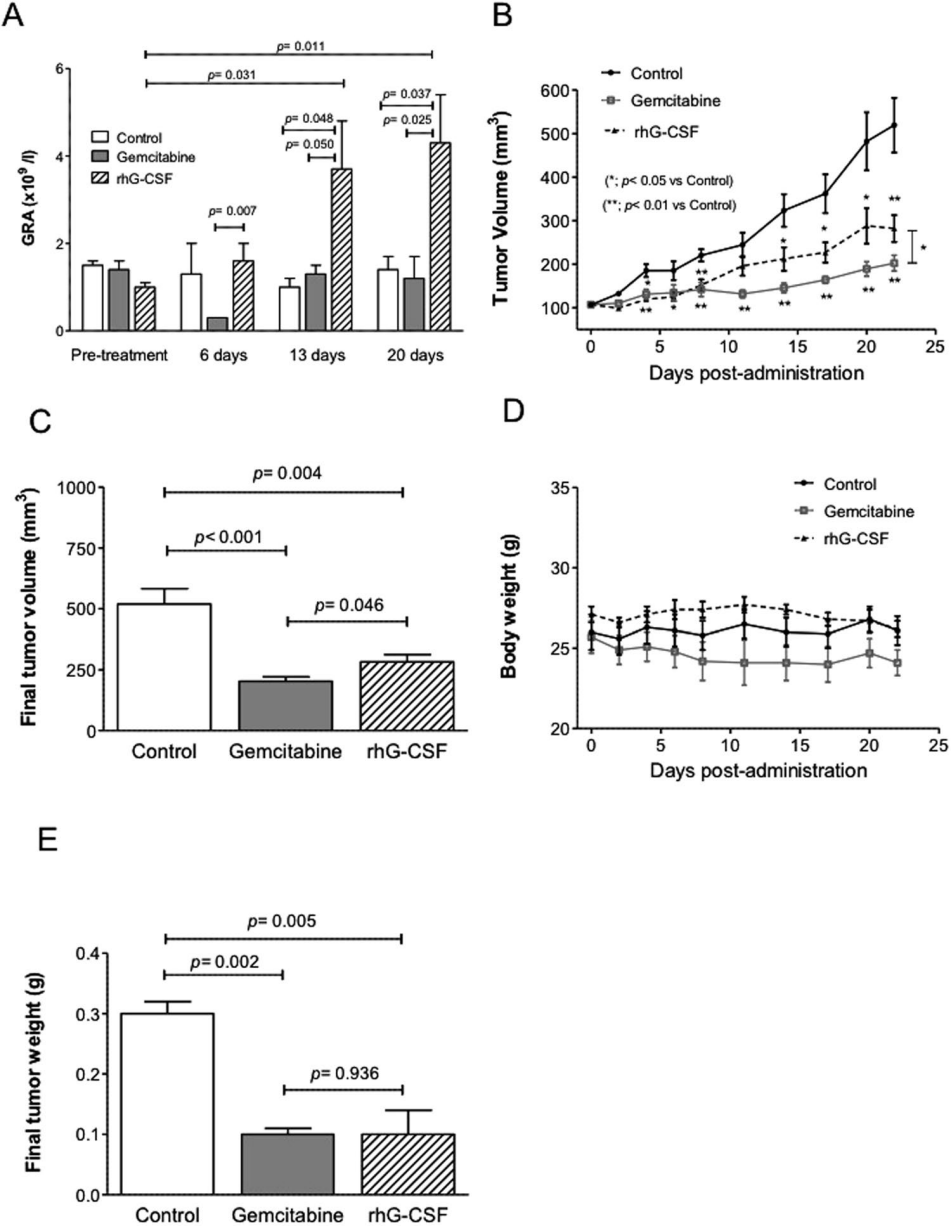
*In vivo* antitumoral activity of rhG-CSF in an xenograft model of pancreatic adenocarcinoma. (**A**) Increase in granulocyte cell count (GRA) induced by rhG-CSF. (**B**) Tumor volume plotted against time for the different treatments. Error bars represent SEM. (**C**) Final tumor volumes for the different treatment groups (**D**). Mean daily tumor weight for each group plotted against days of treatment. (**E**) Final tumor weights. Error bars represent SEM. All differences were assessed using the Mann-Whitney U test.

To monitor the effects of these different treatments on blood cell counts, weekly blood samples were taken. At days 13 and 20, the granulocyte counts of the rhG-CSF-treated mice were three times those of the control and gemcitabine-treated groups (Fig. 1A). Neither monocyte nor lymphocyte counts were affected by rhG-CSF treatment but were reduced with the gemcitabine treatment (data not shown).

The antitumoral response of the tested agents was determined by measuring the change in tumor volume over time. Both the rhG-CSF and gemcitabine treatments led to significant tumor growth suppression by day 4 compared to controls (p < 0.001 and p = 0.013 respectively) (Fig. 1B); this size reduction was maintained until the end of the experiment (day 22) when the mice were killed. From a mean starting volume of 106.9 ± 3.1 mm^3^ for all treatment groups, the final mean tumor volumes reached were 519.4 ± 62.9 mm^3^ for the control mice, 282.0 ± 30.7 mm^3^ for the rhG-CSF-treated mice, and 202.6 ± 18.1 mm^3^ for the gemcitabine-treated mice (p = 0.004 and p < 0.001, respectively, vs. control), and p = 0.046 between the therapeutic treatments (Fig. 1C).

To assess the *in vivo* effects of the rhG-CSF and gemcitabine treatments, mouse body weight and behavior were monitored daily. The rhG-CSF treatment was associated with no increase in mortality, weight loss, signs of suffering, or macroscopic abnormalities suggestive of toxicity. However, the gemcitabine-treated animals showed cachexia and fatigue associated with a slight reduction in body weight by day 5; this lasted until the end of the experiment (Fig. 1D).

At necropsy, the final tumor weights in the rhG-CSF and gemcitabine-treated mice were significantly lower than in the controls (0.143 ± 0.029 g and 0.129 ± 0.018 g respectively vs. 0.316 ± 0.017 g [p = 0.005 and p = 0.002 respectively; no significant difference) (Fig. 1E). No difference in final tumor weight was seen between the rhG-CSF- and gemcitabine-treated mice (p = 0.936).

In order to check that the rhG-CSF antitumor effect was not dependent on the cell line, the same schedule and dosage was administered in another xenograft model of pancreatic adenocarcinoma obtained by injection of MiaPaca cells in athymic mice. A significant reduction of tumor growth without any sign of toxicity was observed (Fig. 1 of Supplementary Information). The use of athymic/immunodeficient mice helped to claim that the innate immune response was the unique responsible for the rhG-CSF antitumor effect and eliminated the possibility that this antitumor effect could be guided by T lymphocytes. However, the antitumoral effect of rhG-CSF was also tested in a syngeneic lymphoma model using immunocompetent mice and similar results were obtained (Fig. 2 of Supplementary Information).

**Figure 2:**
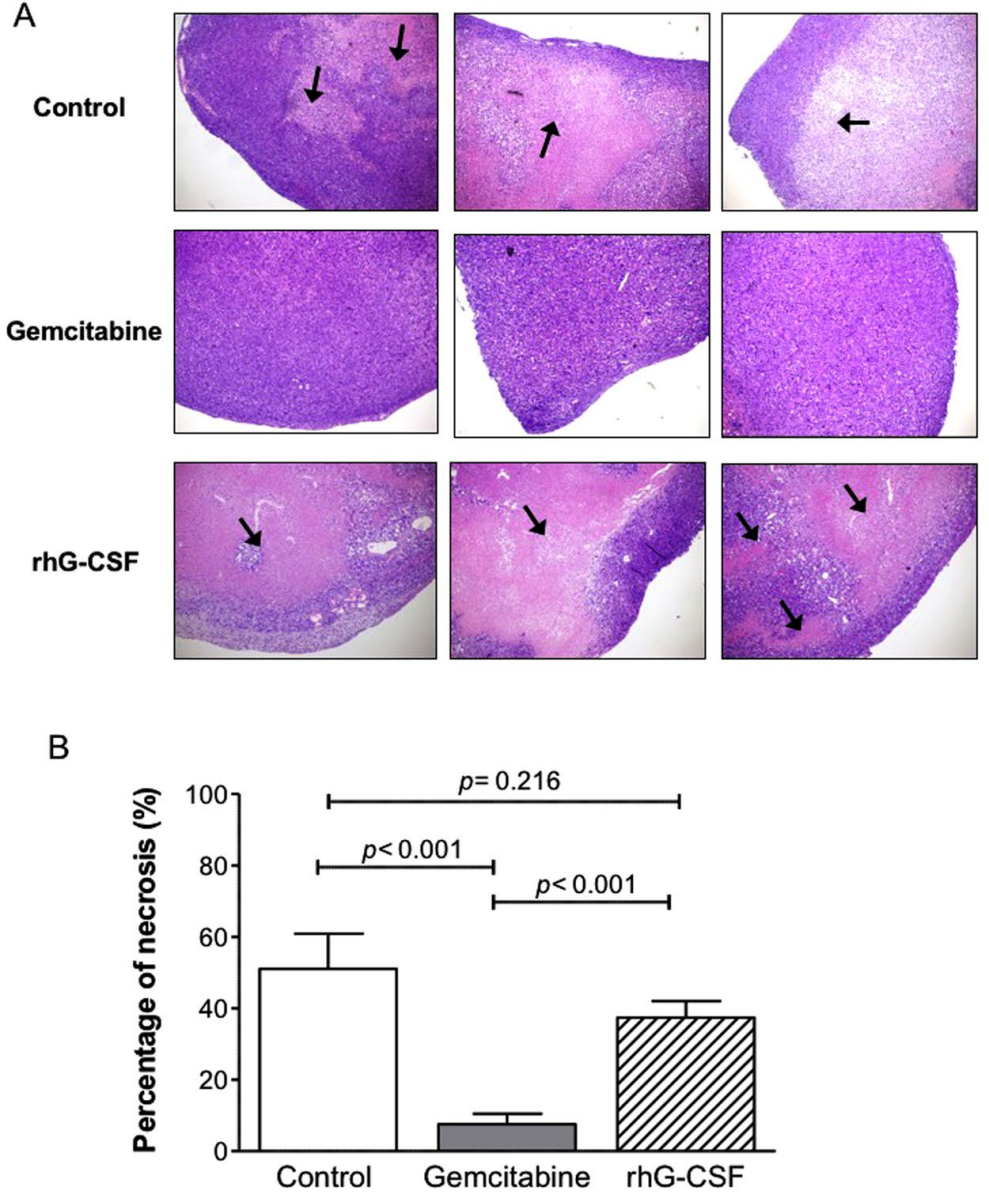
Necrosis induced by the gemcitabine and rhG-CSF treatments. (**A**) H&E staining of three representative tumors from Panc1 xenograft mice treated with saline (control), gemcitabine, or rhG-CSF. Images are shown at their original magnification (x40). Necrotic areas are shown with black arrows. (**B**) Percentage necrosis. Error bars represent SEM. Differences were examined using the Student t test.

### rhG-CSF induced more intratumoral necrosis than did gemcitabine

Tumors treated with rhG-CSF showed higher percentages of necrosis than those treated with gemcitabine (37.4 ± 4.6% vs. 7.5 ± 3.0%; p < 0.001) (Fig. 2A). However, despite being significantly smaller in volume than the control tumors (as shown in Fig. 1B,C), those of the rhG-CSF-treated mice showed no significant differences to the latter with respect to the percentage of necrosis (37.4 ± 4.6% vs. 51.0 ± 9.9, respectively; p = 0.216) (Fig. 2B).

### The spatial distribution of necrosis differed between rhG-CSF and gemcitabine-treated tumors

**Figure 3:**
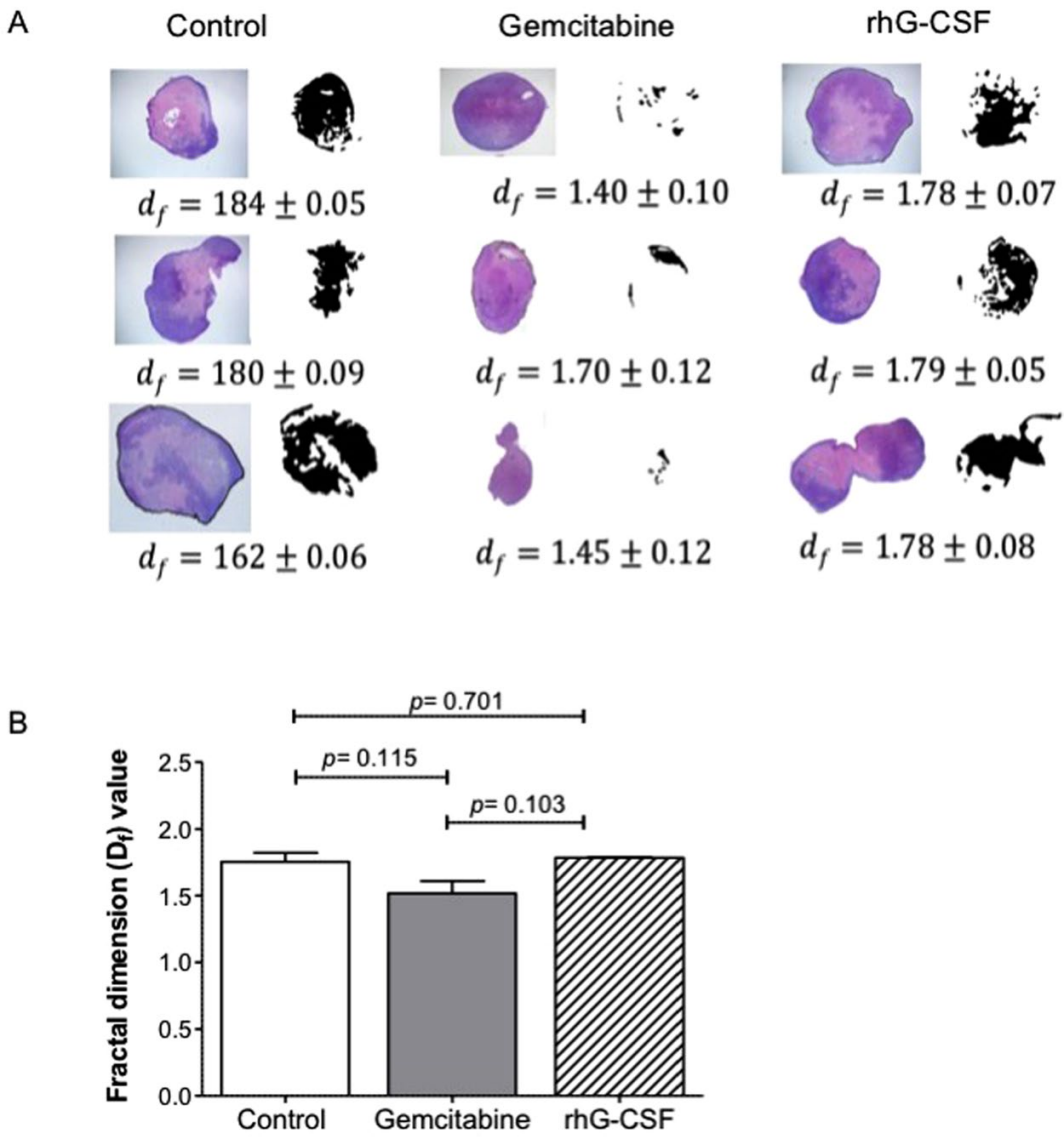
Distribution pattern of necrosis induced by gemcitabine and rhG-CSF. H&E staining of three representative tumors from Panc1 xenograft mice treated with saline (control), gemcitabine or rhG-CSF, plus images showing the necrotic area (shown in black). The fractal dimensions (d_f_) of the necrotic regions were determined for each tumor. Results are expressed as Mean ± SEM. (**B**) Differences in d_f_ between groups. Error bars represent SEM. Statistical analysis was performed using the Student t-test.

The tumors of the control and rhG-CSF-treated mice showed more compact necrosis in their innermost region than seen for the gemcitabine-treated mice, in which the pattern of necrosis was more disperse and closer to the edge of the tumor. Using fractal dimension analysis to characterize the spatial distribution of this necrosis (Fig. 3A), the control and rhG-CSF-treated tumors were shown to have a similar necrotic pattern (mean d_f_ ± SEM: 1.75 ± 0.1 vs 1.78 ± 3 × 10^−3^, p = 0.701). However, these d_f_ values differed from that recorded for the tumors of the gemcitabine-treated mice (1.52 ± 0.1; p = 0.115 and p = 0.103 respectively) (Fig. 3B).

### Proliferation was confined to the external part of the tumors in all treatments

**Figure 4:**
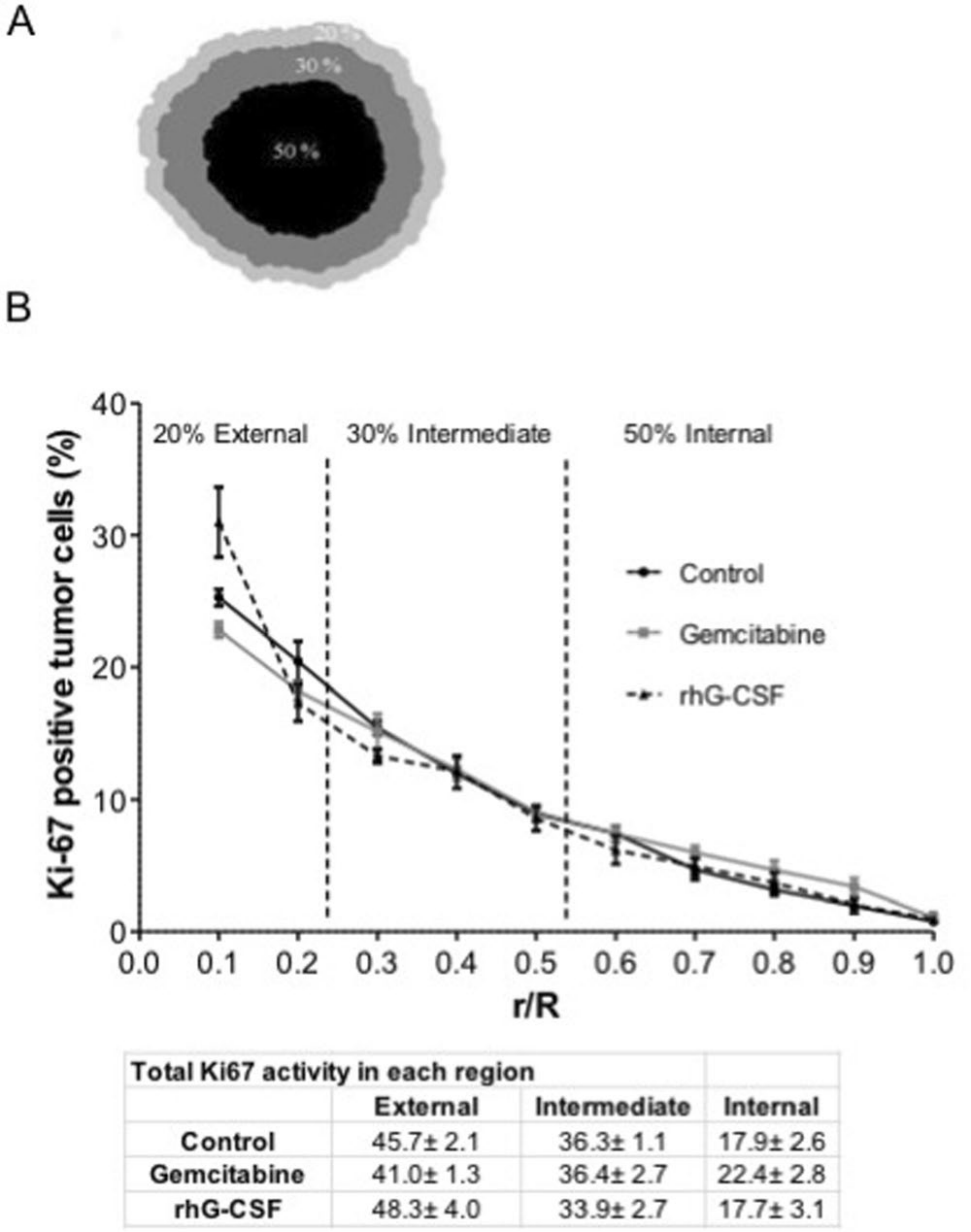
Distribution of active cells inside tumors treated with gemcitabine or rhG-CSF. (**A**) Outer, intermediate and inner regions of the tumor, representing 20% 30% and 50% of the tumor cross-sectional area respectively. (**B**) Percentage of Ki67- positive cells in each tumor region after treatment with gemcitabine or rhG-CSF. Results are expressed as mean percentages ± SEM.

The distribution of the proliferatively active cells was examined in three tumor regions (Fig. 4A). In the most external region, the proliferation rate exceeded 40% for all treatments. In the intermediate region, the proliferation rate was around 30%, with no significant differences between treatments. In the most internal region, proliferative activity was at its lowest for all treatments, with no significant differences between treatments (Fig. 4B).

## Discussion

The present results show that the daily administration of rhG-CSF suppresses tumor growth to an extent comparable to that caused by gemcitabine in a xenograft model of pancreatic carcinoma.

To our knowledge, this is the first study to report the antitumoral effect of neutrophilia induced by rhG-CSF alone in a murine model of pancreatic cancer. Previously, however, Tamamori *et al*.^9^ reported a monoclonal antibody to have a stronger antitumoral effect when simultaneously administered with human rhG-CSF. In addition, neutrophils have been described as responsible for the antitumoral activity seen upon the inoculation of tumors with bacterial lysates^10^ or live bacteria^11^, and for the suppression of tumor growth and metastasis associated with MUC5AC gene knockdown^12^ in murine pancreatic models. Moreover, rhG-CSF has been reported to have an antitumoral effect both alone and in association with other therapies in animal models of other types of cancer, and in all cases this effect has been clearly linked to an increased number of circulating and/or tumor-infiltrating PMNs. For example, the administration of rhG-CSF alone inhibited the formation of metastases in murine melanoma, lung cancer and lymphoma models^13^. rhG-CSF treatment combined with total body hyperthermia achieved an antitumoral effect greater than that seen with either treatment alone in a rat hepatoma model^14^. rhG-CSF plus the monoclonal anti-CD52 antibody (Alemtuzumab) increased survival from 60% to 100% at 12 weeks in a mouse lymphoma model^15^. In an animal model of Ehrlich tumor, rhG-CSF treatment induced strong tumor necrosis and even eliminated some tumors^6^. Increasing the number of circulating PMNs is also reported to boost the efficacy of photodynamic therapy in a rhabdomyosarcoma model^7^, and the importance of increasing the number of circulating PMNs has been well demonstrated when seeking the elimination of melanoma and Walker 256 carcinoma in rats^16,17^.

Evidence of the antitumoral activity of PMNs has also been gathered in humans. The intratumoral administration of bacillus Calmette–Guérin (BCG) in human bladder cancer is one of the best examples of the potential of immunotherapy: high numbers of circulating PMNs correlated with a reduced recurrence of BCG-treated bladder tumors^18,19^. In a randomized, double-blind, placebo-controlled trial of head and neck squamous cell carcinoma, rhG-CSF treatment increased 5-year survival from 47% (placebo group) to 84%^20^. Importantly, rhG-CSF treatment was reported to cure a patient with terminal hepatocarcinoma^21^ - perhaps one of the most important pieces of clinical evidence gathered to date on the positive antitumoral effect of intense and maintained neutrophilia.

The present work shows that daily administration of rhG-CSF alone induces a significant increase in the number of neutrophils, which is associated with a reduction in tumor volume in a murine model of pancreatic cancer. This reduction was not as great as that achieved with the gemcitabine treatment. However, it is important to remember that tumor volume was monitored using calipers. Thus, the volume measurements for the rhG-CSF treated mice might include both the tumor volume and the possible peritumoral inflammation obtained by the induction of neutrophilia; this could have led to overestimation of the true tumor volumes. Indeed, when final tumor weights were compared, the differences between the gemcitabine and rhG-CSF treatments were almost imperceptible. Moreover, intratumoral necrosis was more extensive in rhG-CSF- than in gemcitabine-treated tumors, suggesting that a longer treatment might achieve tumor regression.

Fractal dimension analysis showed the pattern of necrosis in the rhG-CSF-treated tumors to be different from that recorded for the gemcitabine-treated tumors. The pattern for the former tumors was largely localized to the internal part of the tumor, whereas that for the latter tumors was more disperse and external. This is probably because gemcitabine penetrates the tumor, killing it from the inside, while rhG-CSF causes encapsulating neutrophilia which exerts its killing effect from the outside. Indeed, the necrotic pattern for the rhG-CSF-treated tumors, and their distribution of proliferating cells, are fully compatible with the theory of universal dynamics of tumor growth described by Brú *et al*.^8^. In that study, it was shown that the main mechanism of tumor growth is the division and surface movement or ‘diffusion’ of cells at the tumor border, where space is still available. Indeed, space is constantly being made available here via the lytic processes unleashed against the host tissue^22^_._ Inside the tumor, however, where no space is available, the cells become quiescent and eventually necrotic. Based on the results of previous work^6,8^, it was hypothesized that, when rhG-CSF is administered, the resulting intense peritumoral neutrophilia exerts a “pressure effect” that impedes tumor cell diffusion at the tumor surface, impairing the proliferation of tumor cells and causing the cessation of tumor growth. This is accompanied by the eventual internal necrosis of the tumor. Indeed, and in agreement with the results of previous findings^8,23^, the present results revealed proliferative activity to be much greater in the external zone of the tumors (Fig. 4).

It should be noted that the neutrophilia induced by the rhG-CSF treatment was associated with no sign of toxicity, while the gemcitabine-treated mice showed cachexia and fatigue associated with a slight loss of body weight. Unlike the severe adverse effects associated with the use of gemcitabine in humans^24^, rhG-CSF is a very safe drug with minimal side effects^25^. In addition, sustained neutrophilia seems to be a well-tolerated treatment^20^, although this requires formal clinical confirmation. Nonetheless, the present results suggest that rhG-CSF treatment for pancreatic cancer in humans could be effective and safe.

In conclusion, this is the first study to report the antitumoral effect of rhG-CSF alone in a murine model of pancreatic cancer. This treatment increased the granulocyte count without inducing any sign of toxicity. Although a modest study, the results are promising enough to suggest that enhancing the innate immune response via rhG-CSF treatment might be beneficial against pancreatic cancer, and perhaps other tumors.

## Materials and Methods

### Cell line and compounds

Human pancreatic adenocarcinoma Panc-1 cells (CRL-149, ATCC, Virginia, USA) were cultured in DMEM supplemented with 10% fetal bovine serum, 1% glutamine, 100 units/mL penicillin/streptomycin (Life Technologies, Carlsbad, CA, USA), and incubated at 37 °C in a humidified atmosphere containing 5% CO_2_. Gemcitabine (38 mg/ml) (Hospira, Maidenhead UK) and rhG-CSF (Neupogen) (Amgen, Thousand Oaks, CA, USA) were obtained from the Pharmacy Department of Hospital de la Santa Creu i Sant Pau.

### Animals

Female athymic Swiss *nu/nu* mice (Harlan Laboratories Inc., Italy) aged 4–6 weeks weighing 18–20 g, were housed in individually ventilated cages at 21–23 °C and 40–60% humidity, and under a 12 h light-dark cycle. All mice were allowed free-access to an irradiated diet and sterilized water. Animal procedures were reviewed and approved by the Hospital Sant Pau Animal Ethics Committee and the regional Institutional Animal Care and Use Committee.

### *In vivo* experiments

Panc1 cells were injected subcutaneously (20 × 10^6^ cells per flank) into two mice to produce donor tumors. Once grown to 100–120 mm^3^, these donor tumors were cut into fragments of 2–3 mm^3^ and implanted subcutaneously into host mice (as previously described^26^) that had been randomized into three groups: (1) rhG-CSF treatment (n = 7), involving daily administration of subcutaneous rhG-SCF (100 μg/kg in 0.5 mL mm^3^ of saline); (2) intraperitoneal gemcitabine treatment (n = 7) (120 mg/kg every two days in 0.5 mL mm^3^ of saline); and (3) control group (n = 6), involving the subcutaneous administration of 0.5 mL mm^3^ of saline every day. No differences were seen in the initial mean tumor dimensions or weight, or mouse body weight, for each group. Body weight and tumor volumes were recorded twice per week. Tumors were measured using a caliper and volumes estimated according to the formula V = (a.b^2^)/2, (*a*: length or longest diameter; *b*: width or shortest diameter). Measurement began on the first day of treatment (day 0) and finished on day 22 (when the first tumor in the control group reached 1.5 cm in diameter), at which time all mice were sacrificed. All tumors were then removed, fixed in formalin, and paraffin-embedded for histopathological and immunohistochemical studies.

### Blood collection and hematological measurements

A weekly 150 μl blood simple was collected in EDTA tubes (Aquisel K3E, Barcelona, Spain) from three mice per group via intramandibular plexus puncture. Hematological analyses were performed using an automated hematology analyzer (Abacus junior vet, Practice CVM, Navarra, Spain) according to the manufacturer’s specifications and using mouse-specific algorithms (Abacus Software, v.1.21). The variables determined included leukocyte (WBC), lymphocyte (LYM), granulocyte (GRA) and monocyte (MYD) counts.

### Quantification of tumor necrosis and proliferation rates

Paraffin-embedded tissue sections of Panc1 tumors were stained with hematoxylin and eosin (H&E) or Ki67 (DAKO, Carpinteria, CA, USA), a molecule that highlights proliferating cells. Immunohistochemical reactions were performed in a DAKO Autostainer Link48 following the manufacturer’s instructions. All slides were viewed using an Olympus BX15 microscope. Images were acquired using an Olympus DP72 digital camera and processed with Olympus Cell D Imaging 3.3 software (Olympus Corporation, Tokyo, Japan); the final resolution was 1.3 microns/pixel.

Percentage intratumoral necrosis was determined by image processing using verified in-house software^8^. To characterize the spatial distribution of necrosis inside the tumors, the fractal dimension (d_f_) was calculated using the box counting method, using the same in-house software.

The proliferation rate - determined as the percentage of Ki67-positive cells (brown nuclear staining) over the total number of tumor cells - was calculated for three tumor regions: an external region accounting for 20% of the total cross sectional area, an intermediate region accounting for 30%, and an inner region accounting for 50%^8^.

### Statistical analysis

Data were expressed as means ± standard errors. Data sets consisting of more than two groups were analyzed by one-way ANOVA. Differences between means were assessed using the Student t test or Mann Whitney U test as required. All analyses were performed using GraphPad Prism v5.02 (GraphPad Software Inc.).

## Supplementary information

Antitumor effect of rhG-CSF in other mouse models of cancer

## References

[1] Schneider G, Schmid RM (2003). Genetic alterations in pancreatic carcinoma. Mol Cancer..

[2] Altwegg R (2012). Second-line therapy for gemcitabine-pretreated advanced or metastatic pancreatic cancer. World J Gastroenterol..

[3] Huguet F, Mukherjee S, Javle M (2014). Locally advanced pancreatic cancer: the role of definitive chemoradiotherapy. Clin Oncol (R Coll Radiol)..

[4] Balkwill F, Mantovani A (2001). Inflammation and Cancer: back to Virchow?. The Lancet.

[5] Souto JC, Vila L, Brú A (2011). Polymorphonuclear neutrophils and cancer: intense and sustained neutrophilia as a treatment against solid tumors. Med Res Rev.

[6] Brú A, Albertos S, López García-Asenjo JA, Brú I (2004). Pinning of tumoral growth by enhancement of the immune response. Phys Rev Lett.

[7] De Vree WJA (1996). Evidence for an important role of neutrophils in the efficacy of photodynamic therapy *in vivo*. Cancer Res.

[8] Brú A, Albertos S, Subiza JL, López García-Asenjo JA, Brú I (2003). The universal dynamics of tumor growth. Biophys J.

[9] Tamamori Y (2002). Granulocyte-colony stimulating factor enhances chimeric antibody Nd2 dependent cytotoxicity against pancreatic cancer mediated by polymorphonuclear neutrophils. Int J Oncol.

[10] Linnebacher M, Maletzki C, Emmrich J, Kreikemeyer B (2008). Lysates of S. pyogenes serotype M49 induce pancreatic tumor growth delay by specific and unspecific antitumor immune responses. J Immunother.

[11] Maletzki C, Linnebacher M, Kreikemeyer B, Emmrich J (2008). Pancreatic cancer regression by intratumoral injection of live Streptococcus pyogenes in a syngenic mouse model. Gut.

[12] Hoshi H (2013). MUC5AC protects pancreatic cancer cells from TRAIL-induced death pathways. Int J Oncol.

[13] Matsumoto Y (1991). Recombinant human granulocyte colony-stimulating factor inhibits the metastasis of hematogenous and non-hematogenous tumors in mice. Int J Cancer.

[14] Kokura S (1992). Role of polymorphonuclear leukocytes (PMN) and active oxygen species in hyperthermia-antitumoral effect of hyperthermia combined with rhG-CSF. Gan To Kagaku Ryoho.

[15] Siders WW (2010). Involvement of neutrophils and natural killer cells in the anti-tumor activity of alemtuzumab in xenograft tumor models. Leuk Lymphoma.

[16] Zivkovic M (2007). Oxidative burst of neutrophils against melanoma B16-F10. Cancer Lett.

[17] Jaganjac M, Poljak-Blazi M, Zarkovic K, Schaur RJ, Zarkovic N (2008). The involvement of granulocytes in spontaneous regression of Walker 256 carcinoma. Cancer Lett.

[18] Saint F (2001). Leukocyturia as a predictor of tolerance and efficacy of intravesical BCG maintenance therapy for superficial bladder cancer. Urology.

[19] Siracusano S (2007). The role of granulocytes following intravesical BCG prophylaxis. Eur Urol.

[20] Su YB (2006). Double-blind, placebo-controlled, randomized trial of granulocyte-colony stimulating factor during postoperative radiotherapy for squamous head and neck cancer. Cancer J.

[21] Brú A, Albertos S, García-Hoz F, Brú I (2005). Regulation of neutrophilia by granulocyte colony stimulating factor: A new cancer therapy that reversed a case of terminal hepatocellular carcinoma. J Clin Res.

[22] Hay, E. D. Cell biology of extracellular matrix, Springer US, ISBN 978-1-4613-0881-2 (1981).

[23] Kim Y (2006). Induction of pulmonary neoplasia in the smoke-exposed ferret by 4-(methylnitrosamina)-1-(3-pyridyl)-1-butanone (NNK): A model for human lung cancer. Cancer Letters.

[24] Oettle H, Stefan P, Neuhaus P (2007). Adjuvant chemotherapy with gemcitabine vs observation in patients undergoing curative-intent resection of pancreatic cancer. A randomized controlled trial. JAMA..

[25] Bonilla MA (1994). Long-term safety treatment with recombinant human granulocyte colony-stimulating factor (r-methugG-CSF) in patients with severe congenital neutropenias. Br J Haematol.

[26] Céspedes MV (2016). Lurbinectedin induces depletion of tumor-associated macrophages, an essential component of its *in vivo* synergism with gemcitabine, in pancreatic adenocarcinoma mouse models. Dis Model Mech..

