# Supplementary material for: Antitumoral effect of maintained neutrophilia induced by rhG-CSF in a murine model of pancreatic cancer
Source: Sci Rep. 2019 Feb 27;9:2879. doi: 10.1038/s41598-019-39805-y (PMC6393423; doi:10.1038/s41598-019-39805-y)
Supplement: Supplementary file 1 — Antitumor effect of rhG-CSF in other mouse models of cancer [file 41598_2019_39805_MOESM1_ESM.pdf]

“Antitumoral effect of maintained neutrophilia induced by rhG-CSF in a murine model of pancreatic cancer”

A. Brú, R. Bosch, MV. Céspedes, S. Carmona-Guedes, E. Pascual, I. Brú and J. C. Souto

Figure 1

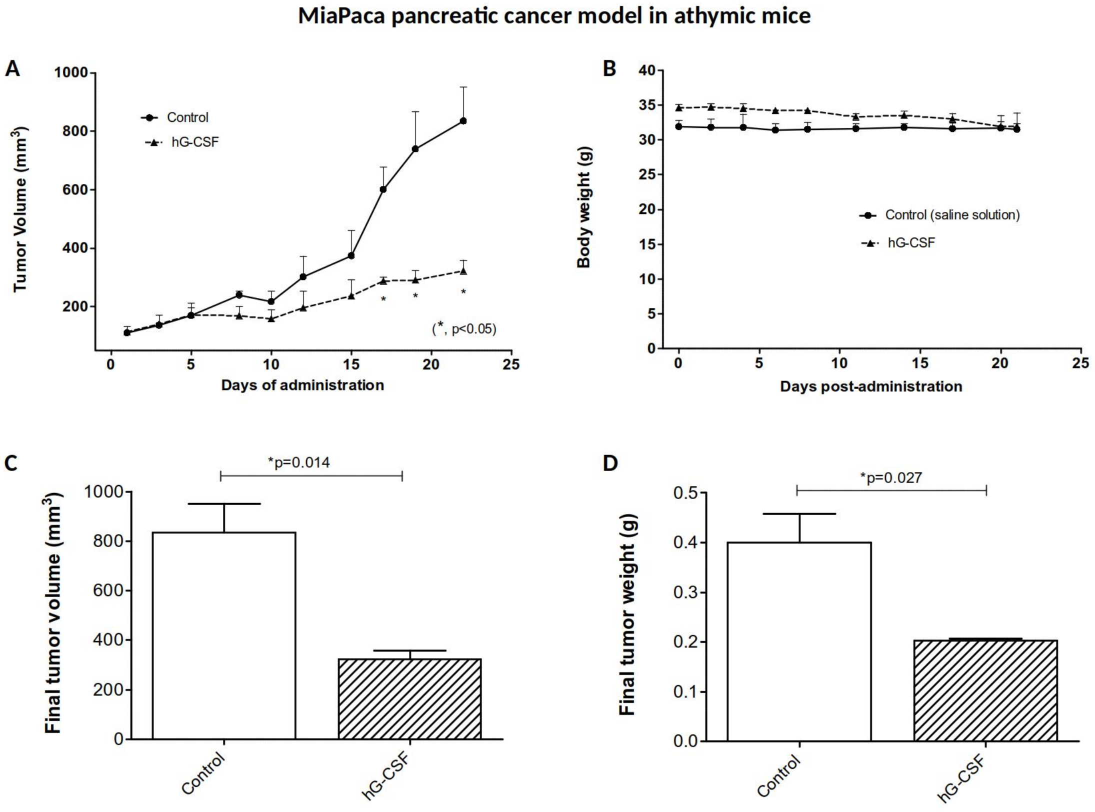

Antitumor effect of hG-CSF in a MiaPaca pancreatic cancer model. MiaPaca cells were injected subcutaneously in 6 athymic nude mice. When tumors reach a volume of approximately 100 mm3, mice were randomized into two groups of 3 mice in order to receive vehicle or subcutaneous hG-CSF 100 µg/kg daily.. After 21 days of treatment all mice were sacrificed. (A) Tumor growth during the experiment. (B) Body mice weight during the experiment. (C) Final tumor volume and (D) final tumor weight. Error bars represent the SEM. *, p<0,05, t-test.

Figure 2

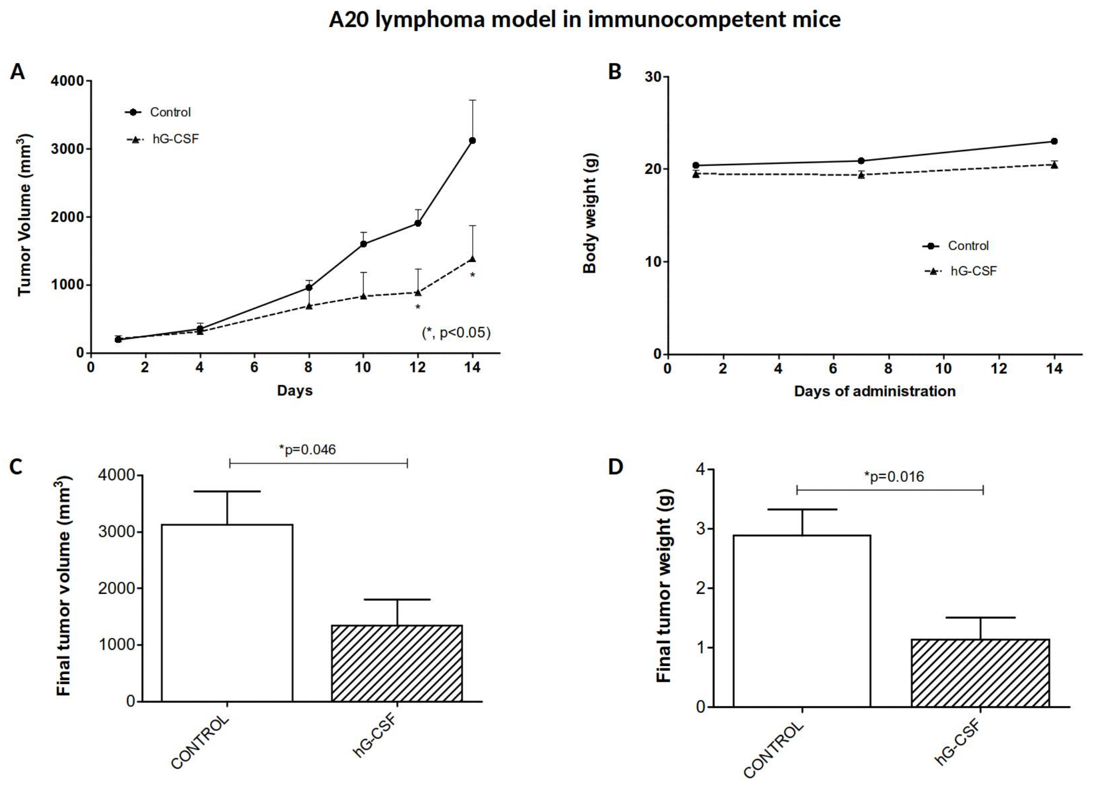

Antitumor effect of hG-CSF in a A20 B-cell lymphoma model. A20 cells were injected subcutaneously in 12 immunocompetent Balb/c mice. When tumors reach a volume of approximately 200 mm3 (one mouse was discarded cause it showed a tumor with an outlier volume), mice were randomized into two groups of 6 and 5 mice in order to receive vehicle or subcutaneous hG-CSF 100 µg/kg daily, respectively. After 14 days of treatment all mice were sacrificed due to the large tumor volume mean of the control group. (A) Tumor growth during the experiment. (B) Body mice weight during the experiment. (C) Final tumor volume and (D) weight. Error bars represent the SEM. *, p<0,05, t-test.
